# Biomarkers for Chronic Pain: Significance and Summary of Recent Advances

**DOI:** 10.1155/2022/1940906

**Published:** 2022-11-07

**Authors:** Sam Eldabe, Ilona Obara, Catherine Panwar, David Caraway

**Affiliations:** ^1^The James Cook University Hospital, Middlesbrough TS4 3BW, UK; ^2^School of Pharmacy and Translational and Clinical Research Institute, The Faculty of Medical Sciences, Newcastle University, Newcastle NE1 7RU, UK; ^3^Panwar Health, Wamberal 2260, Sydney, Australia; ^4^Nevro Corp, Redwood City 94065, USA

## Abstract

Chronic pain can be difficult to predict and a challenge to treat. Biomarkers for chronic pain signal an opportunity for advancements in both management and prevention, and through their research and development offer new insights into the complex processes at play. This review considers the latest research in chronic pain biomarker development and considers how close we are to bringing these from bench to bedside. While some headway has been made that offers efficiencies in patient selection, it is unlikely that a single test will encompass the variety of chronic pain phenotypes. We offer some insights for the near future in biomarker development and areas of continued unmet need.

## 1. Introduction

Pain that persists beyond a reasonable time for healing to take place, usually longer than 12 weeks, is defined as chronic [1]. Considered both a symptom and condition in its own right, chronic pain affects large populations, with around 20% of U.S. adults and 30–50% of adults in the U.K. reporting chronic pain [1–3]. Around 20 million people in the U.S. alone report high-impact chronic pain, which can restrict work, social, and self-care [4, 5]. The burden of chronic pain is therefore not merely clinical, but there is associated with societal impact on productivity, disability, and healthcare utilization [6–11]. Specifically, chronic pain remains a common reason for seeking health care, and despite lacking evidence of long-term effectiveness and guideline warnings, opioids remain a widely used, pain management tool [12, 13]. Providing a means to avoid suboptimal pain management remains a key goal of research [14]. However, even with these efforts, there remains a limited understanding of the fundamental underlying mechanisms of chronic pain [14, 15].

While preclinical models of some chronic pain conditions mimic clinical practice better than others, few biomarkers have widespread utility in clinical practice [15–17]. The complexity and challenges arise from the multiple mechanisms by which chronic pain may occur, compounded by the spectrum of pain that may be influenced by psychological, emotional, and social aspects as well as the environment [15, 17]. Furthermore, the measurement of pain as a symptom remains challenging since it relies on subjective, self-reported pain scale measures [17]. While novel treatments are a major focus of research, accurate biomarkers could unlock the potential of existing therapies and eliminate the reliance on suboptimal therapeutic options and discover new pathways for therapeutic development [15, 17].

Biomarkers are also used in different medical disciplines, to screen for the risk of disease, diagnose, provide a prognosis, and evaluate treatment outcomes [13, 16]. For example, in cardiovascular disease, biomarkers are routinely used in the clinical setting in some cases even at the point of care [18]. However, for chronic pain, biomarkers in practice have been limited to demographic information which may be used to measure risk and estimate prognosis [4]. In chronic pain, the objective of biomarker development has been less about quantifying pain and more about delivering higher-accuracy diagnoses and treatment algorithms based on mechanisms rather than symptoms [13]. Given advances in computing power and artificial intelligence as well as efforts in the understanding of the pathophysiology of chronic pain, this review will discuss the current concepts of biomarkers in the field of chronic pain and how their development could enhance future pain research and management.

## 2. Biomarkers from Bench to Bedside

While the mechanisms behind acute and chronic pain are understood to be quite different, chronic pain remains difficult to manage because of its complexity and following difficulty in identifying and targeting treatments [17]. The complexity of chronic pain relates to the interaction between physical, psychological, and social factors [19]. As a result of the poor understanding of the underlying mechanisms of chronic pain, and perhaps large placebo responses, it may be challenging for new treatments to demonstrate superiority over existing ones in a clinical trial setting [5]. Also, patients who fail conventional medical therapies are labeled as treatment refractory and left with few alternatives [19]. A way to improve these outcomes may come through research centered on the identification of chronic pain-specific biomarkers. In fact, a number of reviews describe the role of biomarker research in chronic pain quite well [5, 13, 15, 17]. Biomarkers are considered to be objective indicators that could be utilized to measure biological processes, pathogenic processes, or intervention responses [13]. Tracey et al. describe the seven different types of biomarkers applicable to the chronic pain field, summarized in Table 1 [17]. Diagnostic biomarkers are used to detect or confirm the presence of a disease [17]. Reckziegel et al. note that such biomarkers should be emergent with chronic pain, yet ideally be reversible with appropriate therapeutic treatment for validation and be able to track variations in chronic pain perception [15]. Monitoring biomarkers assess the status of a disease or the effect of an intervention [17]. Pharmacodynamic/response biomarkers are used to show a biological response following a therapeutic intervention. Predictive biomarkers are used to identify individuals more likely than those without the biomarker to experience an effect following a therapeutic intervention [17]. In this way, Reckziegel et al. also define a subset of predictive biomarkers as preventive, which have mechanisms that reflect consequences between predispositions and injury; this remains an elusive area of research for prevention therapies [15]. Prognostic biomarkers are used to identify the likelihood of a clinical event or progression in patients with a disease of interest [17]. To have value, Reckziegel et al. suggest these biomarkers should focus on pre-existing risks through the acute and chronic phases and should be causally linked to underlying mechanisms of chronic pain, yet for widespread applicability should not differ much between different types of chronic pain [15]. Safety biomarkers can be used to indicate the likelihood or extent of toxicity following a therapeutic intervention [17]. Finally, susceptibility or risk biomarkers can be used to identify the potential for developing a disease [17].

Some authors agree that it is unlikely a single biomarker will be sufficient in clinical practice [5, 13, 15, 17]. Instead, a pain biomarker panel would be more likely to encompass measures from the domains of influence, specifically biological, psychological, social, emotional, and environmental factors. Evaluated carefully and well characterized, biomarkers would not only have the ability to transform care at the bedside, but they could also enhance the characterization of patients in future clinical trials, thereby potentially reducing variability across heterogeneous chronic pain states [5].

## 3. Current State of Play

As reviewed by Davis et al., a growing array of biomarkers is being investigated in chronic pain [5]. These range from electrophysiology of nerves and the brain, to biochemical and “omic” analyses of blood, urine, saliva, cerebrospinal fluid, and other tissues, to structural and functional imaging, and even behavioral analyses and actigraphy [5]. Aside from subjective measurements of pain, research continues into pain response measurement for flare-up and acute pain, including autonomic nervous system changes, measurements such as heart rate variability, skin conductance, and pupil dilation as well as assessment of electrical potential transfer between cells [20]. Research has also now moved into an algorithm approach, where big data sets using multiple inputs can be used to model, measure, and predict chronic pain [20].

### 3.1. Serum-Based Biomarkers

Pro-inflammatory and anti-inflammatory cytokines have been chronic pain biomarker candidates under investigation for over a decade, as reviewed by Nwagwu et al. [21]. In the early days, biomarker discovery was relatively slow, with transcriptome analyses examining the balance of specific gene expression from the peripheral blood of patients with chronic pain [21]. Today, biomarker development is a mixture of panel-based discovery and pathway validation. In investigating biochemical derangement in patients with chronic pain, Gunn et al. analyzed blood and urine biomarker test results from 17,834 unique patients [13]. This analysis revealed a high proportion of patients exhibited at least one abnormal biomarker. In particular, metabolites of the kynurenine pathway (KP), such as quinolinic acid and kynurenic acid, were commonly elevated, suggesting the role of activated KP in chronic pain. Elevated markers of inflammation, such as mercapturic acid, were also identified along with depletion of vitamin B6 and B12-understood to play roles in neurological function as well as evidence of oxidative stress with low levels of serum glutathione [13]. Pain-modulating neurotransmitter metabolites, 5-hydroxyindoleacetate, and vanilmandelate were also depleted in chronic pain states [13]. In support of a disrupted biochemical inflammatory and neurotransmitter state, a systematic review by Kawi et al. determined that exercise was associated with changes in inflammatory and neurotransmitter biomarkers [16].

In summary, further work into the cause and effect of these biochemical pathways is possible through serum analysis, which is relatively easy to acquire from patients on a regular basis.

### 3.2. Urine Metabolites

In the Gunn et al. analysis, a high proportion of patients with chronic pain exhibited at least one abnormal urine biomarker [13]. In a follow-up study, Amirdelfan et al. described the application of a multipanel urinary biomarker assay in patients with chronic pain (the Foundation Pain Index; FPI) across a diverse chronic pain population versus age-matchedpain-free controls [22]. In their analysis, levels of 11 urinary pain biomarkers were measured and evaluated for their correlation with the 36-item Short Form Health Survey (SF-36) [22]. They showed that multibiomarker models provided superior predictive value than single biomarkers in distinguishing chronic pain states versus pain-free controls [22]. Furthermore, the FPI score was able to describe the degree and severity of underlying metabolic derangement that may be driving painful symptomology, therefore providing novel information to complement current subjective assessments [22]. Biomarkers of note included methylmalonic acid, a marker of vitamin B12 status; xanthurenic acid, a biomarker of vitamin B6 status; pyroglutamic acid, a biomarker of glutathione depletion; quinolinic and kynurenine acid which are related to N-methyl-D-aspartate (NMDA) receptors and serotonin synthesis [22]. The research highlights a need for further investigation of the diagnostic utility of the FPI and the impact of the metabolic correction of these pathways is yet to be understood.

In summary, the validity of urine metabolites as markers for chronic pain has not been established, nor is there any evidence that “correction” of the related metabolic pathways has clinical utility. Further improvement in the perception of chronic pain by therapeutic endeavors on the levels of these markers is lacking. However, simple noninvasive screening methods to quantify chronic pain remains a promising goal.

### 3.3. Neuroimaging

Neuroimaging, including structural magnetic resonance imaging (sMRI) and functional MRI (fMRI), and functional near-infrared spectroscopy (fNIRS), has helped us to understand the areas of the brain involved in acute and chronic pain, as well as the relationship between those regions [17, 23]. Neuroimaging has utility right across the spectrum of biomarker uses, as a diagnostic biomarker to response-measuring biomarker and a prognostic biomarker. While it provides valuable information, imaging alone is unlikely to provide a complete understanding of the source, cause, or experience of pain [24]. In terms of its utility as a diagnostic tool, there is debate as to the benefit imaging data offers [24]. While it may provide neuroanatomical and physiological context, the data captured may not explain the whole pain picture, but rather the neural causes and correlates of pain [24]. Modalities, such as sMRI and fMRI, can provide a clearer picture of deeper structures than fNIRS and have been used to identify patients who may respond differently to treatments and as a prognostic tool in selecting patients at high risk of progression or exacerbation [23–25].

The challenge has been that the predictive value of brain imaging for biomarker discovery has been limited to within-person prediction, as brain models differ across individuals [25]. To make use of the data collected and to map it against subjective inputs such as pain scale information, big data, and machine learning algorithms are needed [23]. For example, Zhang et al. summarize a wide range of evoked and resting-state brain imaging biomarker studies, concluding that while many are promising more testing with larger and independent samples are necessary for translational utility [23, 25]. Besides larger sample sizes, combinations of techniques may even be necessary to obtain sufficient sensitivity and specificity as a pain biomarker. In the field of spinal cord stimulation (SCS), De Groote et al. used sMRI to measure changes in brain volume in the hippocampus over time following 10 kHz SCS for failed back surgery syndrome (FBSS). They demonstrated a significant positive correlation between decreased volume in the hippocampus and improved back pain score over time [26]. An fMRI study of the same chronic pain population treated with 10 kHz SCS in FBSS also demonstrated increased connectivity between the affective salience network, regions of the frontoparietal network, and the central executive network, suggesting that 10 kHz SCS influences the affective processing and emotional awareness of pain [27]. Pooling data from different techniques and validating against large, independent samples may help researchers to finally bring MRI biomarkers from the bench to the bedside [23].

### 3.4. Biopotentials

Electrophysiology as a tool for biomarker screening and analysis continues to be explored in investigative studies, though has limited penetration into routine practice to date [5]. Peripheral nerves, neuronal cells, and brain measures including electroencephalogram (EEG) are areas of focus, and given its ease of application in the clinical setting [5], EEG shows the most promise. However, unlike sMRI and fMRI, EEG like fNIRS is limited in its ability to provide a clear view of the deeper structures of the brain [23]. The principle for EEG biomarker development focuses on analyzing stimulated changes as well resting-state activity [5]. Primarily, changes in gamma-frequency potentials and theta power have been associated with pain states [5]. In their systematic review, Pinheiro et al. found persisting pain to lead to maladaptive neural plasticity that can be quantitatively measured using EEG [28]. Specifically, they found that studies show increased theta power to be associated with chronic pain at rest in neuropathic pain and migraine and increased alpha power at resting states in neuropathic pain [28]. They also found disruption in early and late-latencyevent-related potential (ERP) as a result of sensory inputs, including visual stimuli that may be related to attention responses [28]. While cognitive dysfunction is measurable through EEG responses via patterns captured in response to auditory and visual stimuli, debate remains on whether cognitive dysfunction in pain is a consequence or a confounding factor [28]. Quantitative EEG (qEEG) may be able to document responses during treatment with analgesics, which measure neurological efficacy that may not be measurable using standard EEG devices or confounded by other factors, such as psychological state [5].

Changes in alpha power and ERP amplitude have already been used to evaluate pain interventions in patients with neuropathic pain [28]. Telkes et al. demonstrated the application of 10-channel EEG in patients during SCS surgery [29]. Using EEG to evaluate 60 Hz tonic vs. 10 kHz SCS in the intraoperative setting, they could distinguish 10 kHz SCS from SCS OFF state and from tonic stimulation in patients under general anesthesia [29]. Specifically, the relative alpha band power in the primary somatosensory cortex (S1 region) was increased with 10 kHz SCS relative to the SCS OFF state and tonic stimulation [29]. Furthermore, these authors also showed a significant positive correlation between improvement in Oswestry disability index (ODI) scores (from preoperative to postoperative) and change in alpha/theta peak power ratio in the frontal and somatosensory following 10 kHz SCS treatment. This may be another tool for pain relief personalization, giving physicians the ability to screen patients for the likelihood of success prior to SCS implantation.

## 4. Challenges in Adoption

For biomarkers to be clinically useful, they need to be specific, accessible, and also scalable. The first hurdle biomarkers of chronic pain face are interpretation, as the underlying mechanisms of chronic pain can vary widely and are poorly understood in general. A traditional systems biology approach would attempt to quantify a molecule or pathway's role. In chronic pain, the system is multifactorial, making this process more complex than in other conditions. A holistic view of a molecule or pathway ideally includes the pathway from start to finish, which in the case of a biomolecule means from gene to protein to recycling or for nerve function may include multiple molecules, cells, and states of activation.

At present, many biomarkers are in the exploratory phase and face challenges in their shift from the laboratory to the clinic. The complexity of chronic pain etiologies and mechanisms, the interplay of confounding factors, adaptive changes, and therapeutic and placebo effects pose challenges for biomarker discovery, validation, and implementation [30]. When it comes to blood and other bodily tissue profiling, the biochemical, genetic, and “omics” areas offer an ability to generate huge amounts of data across many samples from both individuals and populations. While our ability to collect a large amount of data in this way is currently easy and sometimes as simple as taking a blood or urine sample, our ability to turn big data into meaningful information has been limited. With improvements and innovations in machine learning, neural networks, and artificial intelligence, researchers are able to understand the complex interplay of factors involved in chronic pain better than before. Rather than searching for a biomarker, research groups are moving towards identifying biomarker signatures made up of composite factors [17]. However, even large panel arrays show us that current limitations in our knowledge of the chronic pain states limit the pace of biomarker validation. For imaging and electrophysiology biomarkers, current techniques are complex and require specialized equipment and trained individuals, as well as well-standardized procedures and analysis for routine use [30]. Large-scale studies with improved “signal to noise” ratio (with respect to external influences and confounding factors) are required in large numbers of patients across multiple pain states, in both resting and provoked states, to adopt the application of imaging and electrophysiology biomarkers.

The human experience of chronic pain is unique and personal with multidimensional inputs that vary in contribution to that specific individual's presentation. Even with population-validated biomarkers, patient input that personalizes modification of the therapy seems necessary to optimize therapeutic pain interventions.

## 5. Future Research

The ability to predict the transition from acute to chronic pain and from therapy nonresponder to responder has the potential to change the way patients with chronic pain are managed. Several commercial companies experienced in biomarker analysis are currently exploring ways to bring biomarker screening to the clinic. Several research groups are working on making monitoring easier to implement at the point of care. For example, Cloud-based, mobile device-driven technologies such as those by PainQx are working on making mobile EEG possible at the point of care [31]. Another start-up, MindX, is working on launching commercially available biomarker screening for chronic pain based on the work by Niculescu et al. [32]. Other groups are looking to take advantage of wearable technology, with Evidation Health collecting patient-reported data, alongside wearable and environmental data, from large pain cohorts [33].

## 6. Conclusions

Biomarkers play an important role in health monitoring and decision-making for many conditions, including chronic disease. Chronic pain remains one area where the ability to predict and manage the condition is limited by our currently limited understanding of the underlying mechanisms and its complexity as well as limited treatment options. Investment in biomarker research has the potential to impact care at multiple stages, including susceptibility screening, diagnosis, prognosis, and more. However, given the heterogeneity in presenting phenotypes, there is unlikely to be a silver bullet, a single test for chronic pain. Rather, clinically useful tools are more likely to be composite biomarkers that consist of several measurements. While blood and urine remain easy to sample, relatively few biomarkers have been characterized to date that are useful for routine employment in the clinic. Similarly, while more complex testing such as MRI or EEG provides more specific information about the pathways involved in chronic pain, their application to everyday practice is limited and confounded by other factors that have been discussed above. Already, multifactorial assays derived from algorithm-based analyses of chronic pain datasets are correlating not only with the presence or absence of pain but also with its intensity. A once seemingly impossible task is perhaps a few steps closer to a routine test for chronic pain.

## Figures and Tables

**Table 1:** Biomarker classifications.

Type
(1) Diagnostic
(2) Monitoring
(3) Pharmacodynamic/response
(4) Predictive
(5) Prognostic
(6) Safety
(7) Susceptibility/risk

Adapted from Tracey et al. [17].
